# Domain Engineering in Bulk Ferroelectric Ceramics via Mesoscopic Chemical Inhomogeneity

**DOI:** 10.1002/advs.202200998

**Published:** 2022-04-17

**Authors:** Hao‐Cheng Thong, Zhao Li, Jing‐Tong Lu, Chen‐Bo‐Wen Li, Yi‐Xuan Liu, Qiannan Sun, Zhengqian Fu, Yan Wei, Ke Wang

**Affiliations:** ^1^ State Key Laboratory of New Ceramics and Fine Processing School of Materials Science and Engineering Tsinghua University Beijing 100084 P. R. China; ^2^ Beijing Laboratory of Biomedical Materials Department of Geriatric Dentistry Peking University School and Hospital of Stomatology Beijing 100081 P. R. China; ^3^ State Key Laboratory of High Performance Ceramics and Superfine Microstructures Shanghai Institute of Ceramics Chinese Academy of Sciences Shanghai 200050 P. R. China

**Keywords:** ceramic, chemical engineering, domain engineering, ferroelectric

## Abstract

Domain engineering in ferroelectrics endows flexibility for different functional applications. Whereas the domain engineering strategy for single crystals and thin films is diverse, there is only a limited number of strategies for bulk ceramics. Here, a domain engineering strategy for achieving a compact domain architecture with increased domain‐wall density in (K,Na)NbO_3_ (KNN)‐based ferroelectric ceramics via mesoscopic chemical inhomogeneity (MCI) is developed. The MCI‐induced interfaces can effectively hinder domain continuity and modify the domain configuration. Besides, the MCI effect also results in diffused phase transitions, which is beneficial for achieving enhanced thermal stability. Modulation of chemical inhomogeneity demonstrates great potential for engineering desirable domain configuration and properties in ferroelectric ceramics. Additionally, the MCI can be easily controlled by regulating the processing condition during solid‐state synthesis, which is advantageous to industrial production.

## Graphical abstract

A novel domain engineering strategy is developed for ferroelectric ceramics based on the control of mesoscopic chemical inhomogeneity. Since the strategy can be easily realized via processing control, it is highly accessible to the community. This method is expected to be applicable to various ferroelectric ceramics, offering a valuable opportunity to engineer their macroscopic properties in an unexplored way.

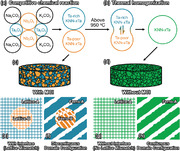

## 1 Introduction

Ferroelectric materials are smart functional materials that are widely used in telecommunication, energy storage, memory, sensing, and actuating devices. They are not only the basis of modern civilization but also critical components of the technology advancement. Ferroelectrics are featured with electrically switchable spontaneous polarization, which forms during the phase transition from a high‐symmetry para‐phase to a low‐symmetry ferro‐phase. Owing to the energy degeneracy of multiple possible polarization orientations, ferroelectrics will spontaneously form a unique domain configuration, where domains with different polarization orientations are separated by domain walls. Since the properties of ferroelectrics depend on the domain configuration, research on domain engineering^[^ ^1^ ^]^ (e.g., domain orientation and domain size) and domain‐wall engineering^[^ ^2^ ^]^ (e.g., domain‐wall mobility, topological structure, and domain‐wall conduction) has attracted extensive interest.

The purpose of domain/domain‐wall engineering in ferroelectrics varies from material to material, for which, different processing techniques are required. For example, domain configurations in single crystals are sensitive to the poling conditions. Rhombohedral relaxor ferroelectric P(Mg_1/3_Nb_2/3_)O_3_‐PbTiO_3_ (PMN‐PT) poled along [001]_c_ directions with a multiple‐domain state shows much larger longitudinal piezoelectric constant *d*_33_ than the one poled along [111]_c_ directions with a single‐domain state.^[^ ^3^ ^]^ Poling PMN‐PT with an alternating‐current electric field was found beneficial for obtaining higher piezoelectricity and optical transparency.^[^ ^1a^ ^]^ Periodically poled LiNbO_3_ with tunable domain widths are useful for electro‐optic applications.^[^ ^4^ ^]^ Different from single crystals, domain configurations in thin films are generally governed by the depolarizing field and mechanical clamping effect.^[^ ^5^ ^]^ Therefore, choices of substrates and modulation of film thickness are decisive to the domain configuration of grown thin films. Over the past decade, numerous fascinating topological structures have been constructed in thin films.^[^ ^6^ ^]^

Compared to single crystals and thin films, the number of engineering approaches in bulk ceramics seems to be limited. The grain‐size effect has been one of the most effective strategies for bulk ceramics.^[^ ^7^ ^]^ Non‐ferroelectric grain boundaries among misoriented grains serve as natural barriers for hindering domain continuity.^[^ ^8^ ^]^ Grain‐size dependence of domain size has been experimentally observed.^[^ ^9^ ^]^ To compensate for the varying elastic and electrostatic energies rise upon paraelectric‐to‐ferroelectric phase transition inside grains of different sizes, miscellaneous combinations of 180° and non‐180° domains will form. As grain size decreases from tens of micrometers to ≈1 µm in BaTiO_3_ ceramic, increased 90° domain wall density and facilitated domain wall mobility result in the enhancement of extrinsic contribution of electric susceptibility (e.g., *d*_33_ up to ≈500 pC/N).^[^ ^10^ ^]^ Similar dependency has been observed in many other ferroelectric ceramics, e.g., (Ba,Ca)(Zr,Ti)O_3_ Pb(Zr,Ti)O_3_, and (Bi,Na)TiO_3_, which is highly favorable for dielectric and piezoelectric applications ^[^ ^11^ ^]^

Since a wide sintering temperature window and high relative density are the prerequisites for achieving the grain‐size effect, the grain‐size effect is not an appropriate strategy for ceramic materials that suffer from poor sinterability, e.g., potassium sodium niobate (KNN).^[^ ^12^ ^]^ KNN ceramics with high Curie temperature and superior electromechanical properties are promising lead‐free piezoelectrics for the next generation.^[^ ^13^ ^]^ Chemical engineering has been the primary strategy to promote the electromechanical properties in KNN.^[^ ^14^ ^]^ Construction of phase boundaries at room temperature via chemical modification is critical to the enhancement, which is explained by the flattened free energy profile that facilitates polarization rotations among coexisting phases.^[^ ^13^ ^]^ Meanwhile, characteristic microstructures, including dense nanodomain,^[^ ^15^ ^]^ slush polar region,^[^ ^16^ ^]^ and local structural heterogeneity,^[^ ^17^ ^]^ have been frequently observed in high‐performance KNN‐based materials. It has been suggested that these characteristic domain configurations might also contribute to the enhanced electrical susceptibility, though the understanding is still at a shallow level. Further enhancement of electrical susceptibility is advantageous to the development of KNN in piezoelectric applications, for which a novel engineering technique is essential.

**Figure 1:**
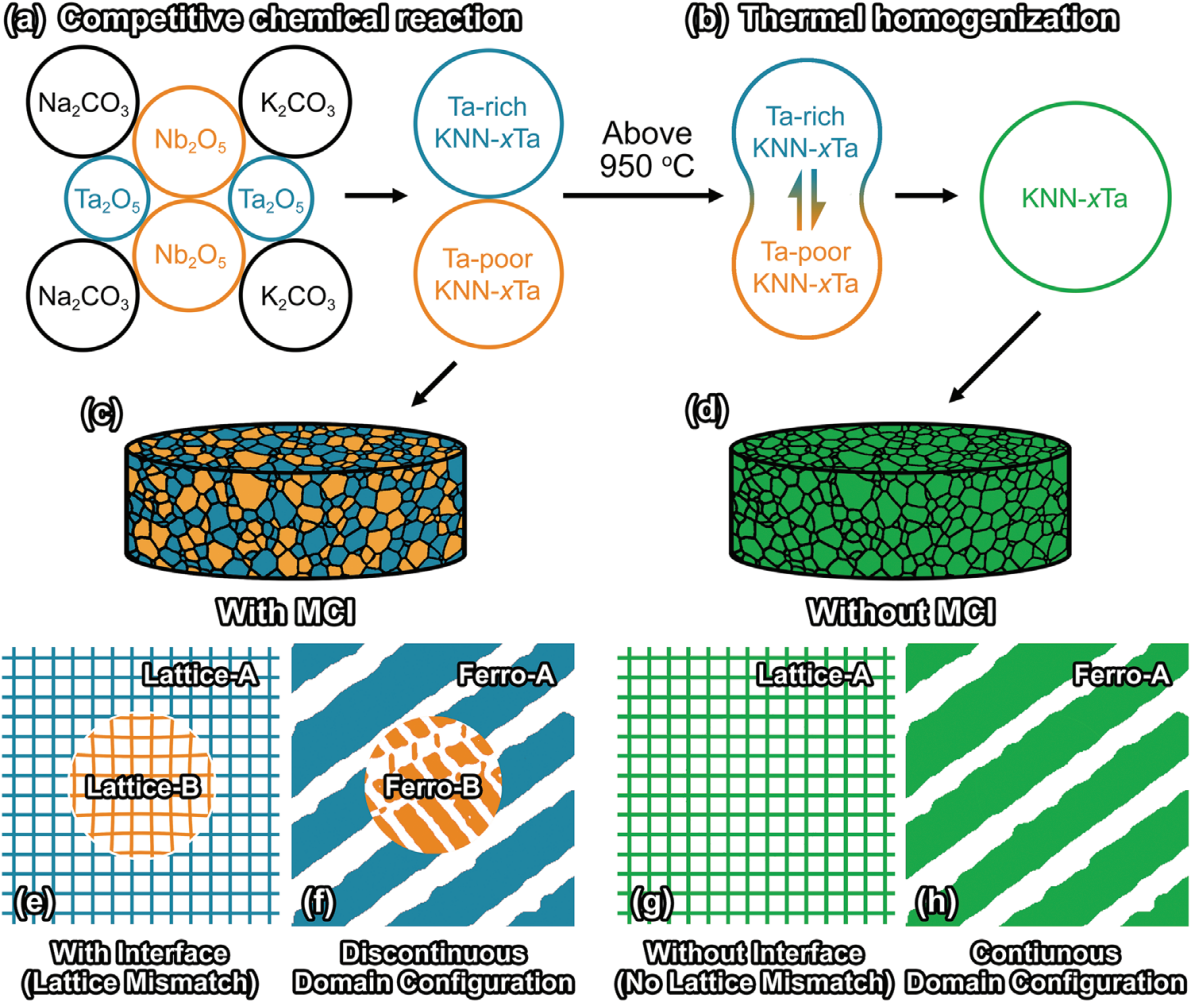
The concept of domain engineering in bulk ferroelectric ceramics via mesoscopic chemical inhomogeneity (MCI). a) Illustration of competitive chemical reaction among raw materials occurs during synthesis and resulted chemical inhomogeneity in synthesized powder. b) Illustration of the chemical inhomogeneity mitigated by thermal homogenization at above 950 °C. Ceramics c) with MCI and d) without MCI sintered from synthesized powders obtained in (a) and (b), respectively. e) Presence of interface in ceramic with MCI. f) Domain configuration is interrupted at the interface. g) No interface form in ceramic without MCI. h) Domain configuration is continuous.

In the present study, we proposed using mesoscopic chemical inhomogeneity (abbreviated as MCI) to engineer the domain configuration in KNN ferroelectric ceramics with fixed chemical stoichiometry (**Figure** **1**). The concept was validated in a material system, i.e., KNN‐Ta solid solution, via wisely controlling the processing conditions during solid‐state synthesis. By utilizing MCI, we successfully break the long‐range domain continuity, decrease domain size, and increase domain‐wall density, as directly evidenced in the piezo‐force microscopy and transmission electron microscopy (TEM) measurements. Meanwhile, significant diffused phase transitions were observed in the sample with MCI, which is favorable for achieving better thermal stability. The formation of the MCI interface was systematically investigated. The impact of the engineered domain configuration on electrical properties was discussed.

## 2 Results and Discussion

### 2.1 Solid‐State Synthesis of Powders

**Figure 2:**
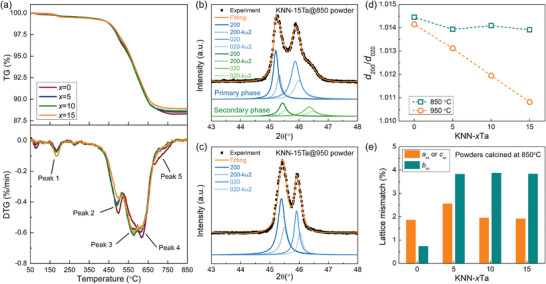
Solid‐state synthesis of KNN‐*x*Ta powders. a) TG loss and DTG of raw powders measured as a function of temperature. Room‐temperature XRD patterns of 200/020 reflections of KNN‐15%Ta powders calcined at b) 850 ℃ and c) 950 ℃. d) Variation of *d*_200_/*d*_020_ in powders calcined at different temperatures. e) The lattice mismatch between primary and secondary phases in the powders calcined at 850 ℃. The labeling of reflections, derived *d*‐spacing, and lattice parameters are based on the symmetry of the monoclinic perovskite subcell.

KNN‐*x*Ta (for *x* = 0%, 5%, 10%, and 15%) powders were prepared by using the solid‐state synthesis (Figure 1a–d). The solid‐state synthesis was analyzed as shown in **Figure** **2**. The thermogravimetric (TG) analysis of the solid‐state synthesis of these powders is shown in Figure 2a. While the derivative of TG (abbreviated as DTG) peak 1 corresponds to the dehydration of carbonates,^[^ ^18^ ^]^ DTG peaks 2–5 correspond to the decomposition of carbonates (i.e., K_2_CO_3_ and Na_2_CO_3_), which only occur when reacting with Nb_2_O_5_ or Ta_2_O_5_.^[^ ^19^ ^]^ The presence of multiple DTG peaks suggests competitive reactions among raw materials occur during synthesis. The competitive reaction can result in a strong chemical inhomogeneity in the synthesized products, depending on the choices of raw materials (e.g., powder size and crystal structure).^[^ ^19^ ^]^ Even though, the chemical inhomogeneity in synthesized products can be vanished to some extent via thermal homogenization at above 950 °C, as discussed in our previous work.^[^ ^19^ ^]^

To demonstrate the effectiveness of the MCI effect, we purposely designed two series of samples by manipulating thermal homogenization. 2 series of KNN‐*x*Ta powders were calcined at 850 and 950 °C, to prepare chemically inhomogeneous and chemically homogeneous powders, respectively. From the XRD patterns shown in Figure 2b,c and Figure S1 (Supporting Information), the presence of two coexisting phases (including primary phase and secondary phase) can be significantly found in powders calcined at 850 °C, but the amounts of secondary phase seem to diminish for the counterparts calcined at 950°C. The coexistence of two phases is a result of the competitive chemical reaction. For KNN‐0Ta, the primary phase and secondary phase are KNN solid‐solution of different K/Na ratios, while for KNN‐*x*Ta (*x* = 5%, 10%, 15%), variation of Nb/Ta ratios will superpose on the K/Na ratios. By analyzing the *d*‐spacing ratio of 200(or 002)/020 (denoted as *d*_200/020_)) reflections of primary phases (Figure 2d), we find that the *d*_200/020_ of powders calcined at 850 °C merely changes but the *d*_200/020_ of powders calcined at 950 °C drastically decreases, supporting the effectiveness of thermal homogenization at 950 °C. The lattice mismatch between the primary phase and secondary phase in powders calcined at 850 °C is roughly estimated to be 1–4% (Figure 2e).

### 2.2 MCI and Domain Configuration

**Figure 3:**
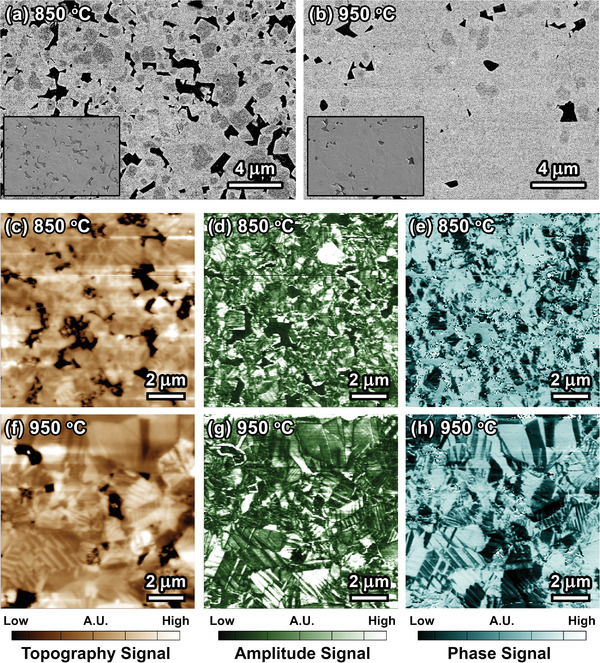
Presence of MCI and interruption of domain configuration in sintered ceramics. SEM‐BSE images of a) KNN‐15Ta@850 and b) KNN‐15Ta@950 ceramics. The inset shows the SEM‐SE images. PFM c) topography, d) amplitude, and e) phase signals of KNN‐15Ta@850. PFM f) topography, g) amplitude, and h) phase signals of KNN‐15Ta@850. The regions of interest measured in PFM are 10 µm × 10µm. The original PFM data with an absolute scale bar can be found in the Figure S5 (Supporting Information).

Sintered ceramic samples from KNN‐15%Ta powders calcined at 850 °C (abbreviated as KNN‐15Ta@850) and 950 °C (abbreviated as KNN‐15Ta@950) were selected for comparison, as the discrepancy of chemical homogeneity is expected to be the most significant. To ensure consistency, the samples were prepared in the same batches and same sintering conditions (i.e., furnace, duration, and temperature). The morphology of sintered ceramics was characterized by using scanning electron microscopy (SEM), as shown in **Figure** **3**. From the back‐scattered electron (BSE) image, strong signal contrast is observed in the KNN‐15Ta@850 sample (Figure 3a), with a characteristic core‐shell heterostructure. The contrast suggests that strong chemical inhomogeneity occurs in the sintered sample. Since Ta is atomically heavier than Nb, Ta can scatter more electrons and result in a stronger BSE signal. Therefore, the “core” and “shell” correspond to the Ta‐poor and Ta‐rich regions. On the contrary, chemical inhomogeneity can also be observed in the KNN‐15Ta@950 sample (Figure 3b) but the area is greatly lessened, suggesting that the sample possesses a better chemical homogeneity. The chemical distributions in both samples were also checked by electron probe microanalysis (EPMA) (Figure S2, Supporting Information). EPMA is one of the best characterization methods for the chemical analysis of ceramic samples since it can provide the analysis at a large scale (e.g., 30 µm × 30 µm), which can characterize tens to hundreds of grains simultaneously, thus giving a statistically meaningful result. The EPMA results were found in good agreement with the SEM‐BSE results. We note a very similar core‐shell heterostructure was obtained early in other Ta‐modified KNN systems.^[^ ^21^ ^]^ Wang et al. suggested that prolonged high‐temperature annealing cannot eliminate the chemical inhomogeneity but utilizing the precursor method can substantially mitigate the issue.^[^ ^21a^ ^]^ However, in the present study, we demonstrate that the chemical homogeneity can be flexibly controlled by simply regulating the calcination temperature.

**Figure 4:**
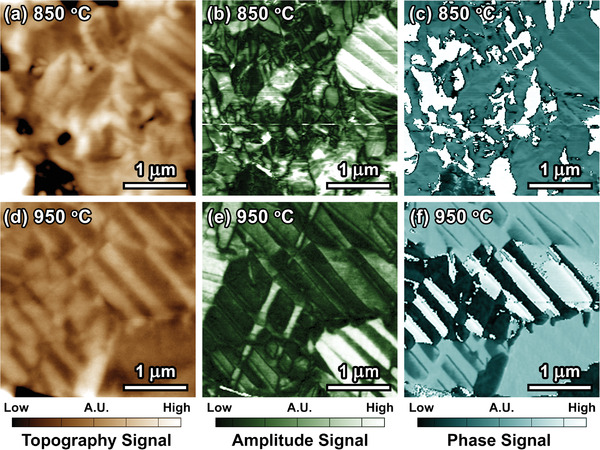
PFM measurement at magnified 3 µm × 3 µm regions. PFM a) topography, b) amplitude, and c) phase signals of KNN‐15Ta@850. PFM d) topography, e) amplitude, and f) phase signals of KNN‐15Ta@950. The original PFM data with an absolute scale bar can be found in the Figure S6 (Supporting Information).

To characterize the domain configuration, piezoresponse force microscopy (PFM) was employed. Unusual domain configuration was found in the KNN‐15Ta@850 sample (Figure 3c–e), where typical long‐range stripe‐like or herringbone‐like domain patterns seem to be missing in this sample, as compared to the KNN‐15Ta@950 sample (Figure 3f–h). However, as we increase the magnification, i.e., measuring a region of 3 µm × 3 µm, a small amount of stripe‐like domains was discovered along with the irregular slush‐like domain patterns (**Figure** **4**a–c). Visually, a compact domain configuration with increased domain‐wall density is obtained in the KNN‐15Ta@850 sample. At specific regions, domains with sizes roughly smaller than 100 nm can be found. By semi‐quantitatively analyzing the edges in phase images using canny detection^[^ ^22^ ^]^ (Figure S3, Supporting Information), the KNN‐15Ta@850 sample was found to possess a higher domain‐wall density than the KNN‐15Ta@950 sample. To verify the ferroelectricity of KNN‐15Ta@850, i.e., the reversibility of polarization, direct writing of domains were performed in PFM measurement, as shown in Figure S4 (Supporting Information). A local coercive voltage found during the domain writing reveals the characteristic of domain switching, which confirms the authenticity of the irregular ferroelectric domain pattern in KNN‐15Ta@850. The SEM‐BSE and PFM results indicate that MCI is the origin of the formation of the unusual domain configuration in the KNN‐15Ta@850 sample.

### 2.3 Interface at MCI Regions

**Figure 5:**
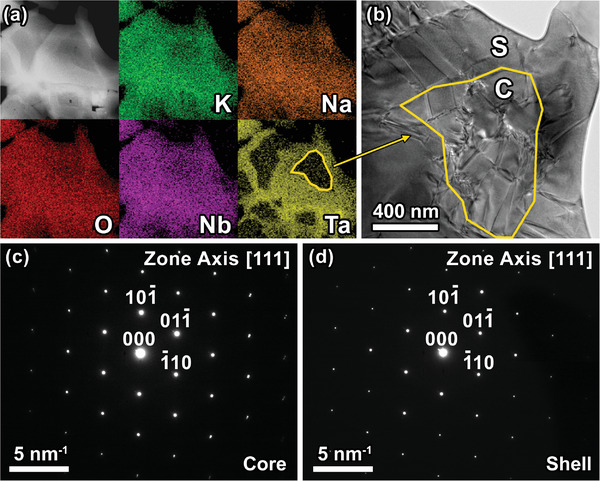
TEM analysis of the MCI regions in KNN‐15Ta@850 sintered ceramics. a) EDS analysis and b) bright‐field TEM image of the MCI region. SAED patterns of c) core and d) shell regions. The letters “C” and “S” represent “core” and “shell” respectively. Please note that the golden‐colored boundary sketched in (b) is only an estimation based on the EDS result, which does not represent the exact interface.

The KNN‐15Ta@850 sample was characterized by using TEM, as shown in **Figure** **5**. By using energy dispersive spectroscopy (EDS; Figure 5a), MCI can be easily observed throughout the whole sample. The EDS result shows a significant inhomogeneous distribution of Ta, where Ta is deficient inside the “core” region. This is in agreement with the SEM‐BSE and EPMA results mentioned earlier. An MCI core‐shell region of sub‐micrometer size (with the size of “core” roughly 800 nm) was chosen for analysis. From the bright‐field TEM image (Figure 5b), stripe‐like and irregular slush‐like domain patterns were again found within the region. Noticeably, many domain walls coincide with the core‐shell interface, which is marked with a golden‐colored boundary. The selected area electron diffraction (SAED) patterns at the “core” and “shell” regions were found nearly overlapping (Figure 5c,d), suggesting an excellent epitaxial relationship across the interface. Still, we notice a tiny tilting angle as shown in Figure S7 (Supporting Information), which indicates a slight misorientation between lattices.

**Figure 6:**
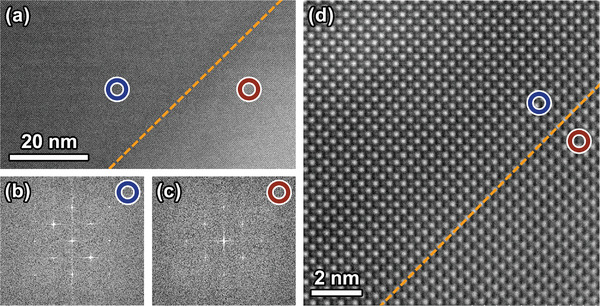
a) HRSTEM image at core‐shell interface. The blue and red circles represent “core” and “shell” regions, respectively. FFT of HRSTEM images at b) “core” and c) “shell” regions. d) Atomic‐resolved high‐angle annular dark‐field image at the interface.

The core‐shell interface was further analyzed by using atomic‐scale imaging, as shown in **Figure** **6**. The fast Fourier transformation (FFT) of the high‐resolution scanning transmission electron microscopy (HRSTEM) image of “core” (Figure 6b) was found nearly identical with that of “shell” (Figure 6c) region, suggesting good epitaxy. From the atomic‐resolution image (Figure 6d), a coherent interface can be observed between “core” and “shell” regions. However, slight lattice misorientation was again found near the interface, which can be inferred from the blurry resolution at the “shell” region (bottom‐right corner). It is surprising to find that a coherent interface forms naturally between ferroelectric phases with different lattice parameters. We conjecture that the mismatch strain between phases has been relieved by either long‐range lattice distortion or the formation of non‐180° domain walls near the interface, which can account for the increased domain‐wall density and the complexity of domain configuration.

We discovered a unique microstructure at another MCI region, as shown in Figure S8, S9 (Supporting Information). At this specific region, a significant variation of the K/Na ratio was observed, along with the Nb‐Ta inhomogeneity. A complex domain pattern with domain sizes of roughly tens of nm was observed inside the “core.” From the SAED result of “core,” reflections of a superlattice were found to coexist with reflections of KNN perovskite. The characteristic microstructure observed in our sample is very similar to the one reported in the previous work.^[^ ^23^ ^]^ The formation of the superlattice in the KNN system has been explained by the tilting of oxygen octahedrons, which is suggested to be associated with the variation of the K/Na ratio.^[^ ^24^ ^]^ The observation suggests that the microstructural modification due to the MCI effect is complicated, and the influence of such microstructure requires further investigation.

### 2.4 Diffused Phase Transition

**Figure 7:**
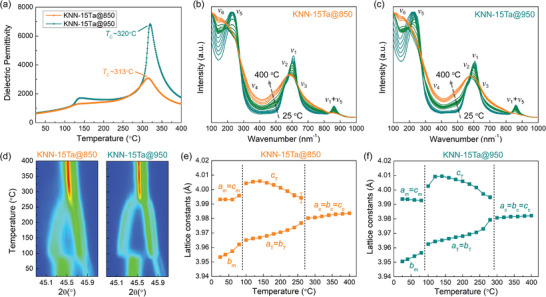
a) Temperature‐dependent dielectric permittivity of KNN‐Ta@850 and KNN‐Ta@950. Temperature‐dependent Raman spectra of b) KNN‐Ta@850 and c) KNN‐Ta@950. The spectra are visually categorized into three groups with different colors, where blue‐colored lines, green‐colored lines, and orange‐colored lines correspond to orthorhombic (or monoclinic), tetragonal, and cubic phases, respectively. d) Temperature‐dependent XRD patterns of 200 reflections of KNN‐Ta@850 and KNN‐Ta@950. Lattice constants of e) KNN‐Ta@850 and f) KNN‐Ta@950 derived from the fitting of 200 reflections. A slight mismatch of phase transition temperatures measured in XRD likely originates from the common inaccuracy of temperature measurement.

The impact of the MCI on the properties of KNN‐*x*Ta samples was investigated, as presented in **Figure** **7** and Figures S10 (Supporting Information). From the temperature‐dependent dielectric spectra (Figure 7a), significant diffused phase transitions were observed in KNN‐15Ta@850. Diffused phase transition has been useful for achieving thermal stability in KNN‐based ferroelectrics.^[^ ^25^ ^]^ In contrast, relatively sharp phase transitions were observed in KNN‐15Ta@950. The diffuseness of phase transition in KNN‐15Ta@850 can be inferred from the *γ* value ≈1.5 obtained from the fitting of modified Curie‐Weiss law (Figure S11, Supporting Information).^[^ ^26^ ^]^ It is known that the *γ* varies within a range from 1 to 2, of which the bottom and upper limits correspond to typical ferroelectric and relaxor, respectively. The *γ* ≈ 1.5 obtained in KNN‐15Ta@850 lies at the middle of the limits, suggesting that the sample is somewhat in between typical ferroelectric and relaxor. From the analysis of inverse of dielectric permittivity, first‐order ferroelectric‐paraelectric phase transition was found in KNN‐15Ta@850 and KNN‐15Ta@950 (see Figure S12, Supporting Information). It is worth noting that KNN‐15Ta@850 shows a stronger first‐order nature.

The phase transitions were investigated by using the temperature‐dependent Raman spectroscopy (Figure 7b,c) since the structural evolution during phase transition can reflect on the change of intensity and shift of the characteristic vibration modes.^[^ ^27^ ^]^ It was observed that the variation of vibration modes with increasing temperature in KNN‐15Ta@850 is almost continuous while that in KNN‐15Ta@950 can be rather easily categorized into three groups, which correspond to the orthorhombic (or monoclinic),^[^ ^20^ ^]^ tetragonal, and cubic phases.^[^ ^27^ ^]^ Meanwhile, the phase transitions were also investigated by using the temperature‐dependent XRD (Figure 7d–f). Diffused and sharp phase transitions were observed in KNN‐15Ta@850 and KNN‐15Ta@950, respectively. We notice that the width of the diffraction peaks in KNN‐15Ta@850 is relatively larger than those in KNN‐15Ta@950 (see Figures S13 and S14 and Table S2, Supporting Information). The observation likely indicates the presence of coexisting ferroelectrics with different lattice parameters, i.e., core and shell, which is in good agreement with the aforementioned results.

Even though, frequency dispersion in the typical relaxor was not observed in KNN‐Ta@850 (see Figure S15, Supporting Information). The absence of frequency dispersion in KNN‐based ceramics with high *γ* has been frequently encountered.^[^ ^16^ ^]^ Therefore, another explanation is required to account for the diffused phase transition in KNN‐Ta@850. We would like to postulate a scenario as follows: for a core‐shell heterostructure, the dielectric properties may be treated as a superposition of two components, i.e., “core” and “shell”.^[^ ^28^ ^]^ Intuitively, we should observe two significant *T*_C_ for a core‐shell heterostructure from the temperature‐dependent dielectric spectra, where “shell” should have a lower *T*_C_ due to the high content of Ta doping. However, this is contradictory to the experimental observation. Since we have confirmed the presence of coexisting ferroelectrics (core and shell) with different lattice parameters but a coherent interface was observed, we conjecture that the core and shell are influencing each other, and they tend to relieve the mismatch strain whenever possible.

Local stress has known to be a decisive factor in the properties of phase transition in ferroelectrics.^[^ ^29^ ^]^ Let us imagine a phase transition of core‐shell heterostructure upon heating: when the temperature increases and becomes higher than the *T*_C_ of “shell” but is still lower than the *T*_C_ of “core”, the “shell” tends to transform into a paraelectric phase while “core” remains its ferroelectric phase. The lattice mismatch between paraelectric “shell” and ferroelectric “core” should become significant and result in considerable mismatch strain (or stress). On one hand, the imposed local stress may prevent the phase transition of “shell”, i.e., restricting the change of its lattice structure; while on the other hand, it may facilitate the phase transition of “core”. The phenomenon will result in an enhanced *T*_C_ for “shell” and a reduced *T*_C_ for “core”. Namely, the ideally two separated sharp *T*_C_ in a core‐shell heterostructure will merge into one but diffused *T*_C_. Schader et al. discovered that applied compressive stress can increase the first‐order nature of ferroelectric–paraelectric phase transition of BaTiO_3_.^[^ ^29a^ ^]^ The increased first‐order nature observed in KNN‐15Ta@850 is likely associated with the imposed local stress due to the formation of the interface. The proposed mechanism can reasonably explain the diffused phase transition and the missing frequency dispersion in KNN‐15Ta@850. We think that the hypothesis could be further examined by using a temperature‐dependent TEM in the future.

Conventionally, to achieve diffused phase transition, one may have to purposely design the chemical compositions.^[^ ^30^ ^]^ Recently, Zheng et al. proposed using a 2‐2 type multilayers composite heterostructure with gradient chemical compositions to induce a diffused phase transition in KNN‐based ceramics to achieve enhanced thermal stability.^[^ ^31^ ^]^ We think the strategy is very similar to the one in this work, but the heterostructure constructed herein is a 0–3 type core–shell structure. In the strategy proposed by Zheng et al., layers of multiple compositions have to be selected and stacked together.^[^ ^31^ ^]^ However, our work demonstrates that diffused phase transition can be easily modulated even in one fixed chemical composition, as long as we can feasibly engineer the chemical inhomogeneity. The simplicity of our strategy would be advantageous for large‐scale manufacturing.

Since chemical inhomogeneity is a general phenomenon in materials, we expect the MCI effect can be achieved in most of the ferroelectric ceramics, but the regulation of processing parameters might have to be further optimized for each composition independently. Modulation of the core‐shell heterostructure, e.g., the core‐shell volume ratio, density of core–shell interface, and degree of lattice mismatch, can be meaningful attempts for achieving different properties.^[^ ^28^ ^]^ The heterostructure is not only advantageous for enhanced thermal stability but also beneficial for potential enhancement of electromechanical properties, where an excellent demonstration can be found in our recent work.^[^ ^32^ ^]^

## 3 Conclusion

In summary, modification of domain configuration via MCI was successfully validated in KNN‐*x*Ta ferroelectric ceramics. The MCI‐induced interface at the core‐shell heterostructure leads to the formation of a complex domain configuration with a smaller domain size and higher domain‐wall density. Meanwhile, a significant diffused phase transition was observed in the KNN‐*x*Ta with core‐shell heterostructure, which is beneficial for enhancing thermal stability. We propose that the complex stress field that arises at the coherent interface between core and shell with different lattice parameters can account for the formation of increased domain‐wall density and the diffused phase transition, which is the key to the domain engineering via MCI. We believe that domain engineering via MCI is highly accessible and beneficial to the community, by which desirable domain configuration and properties can be obtained in ferroelectric ceramics.

## 4 Experimental Section

### Sample Preparation

(K_0.5_Na_0.5_)(Nb_1‐_ *_x_*, Ta*_x_*)O_3_ (abbreviated as KNN‐*x*Ta) (for *x* = 0%, 5%, 10%, and 15%) powders were prepared by the conventional solid‐state reaction method. The raw materials K_2_CO_3_ (>99.0%, Sinopharm), Na_2_CO_3_ (>99.8%, Sinopharm), Nb_2_O_5_ (>99.99%, Sinopharm), and Ta_2_O_5_ (>99.99%, Sinopharm) were weighed accordingly and subjected to planetary ball milling at 300 rpm for 24 h with ethanol as a dispersant. The mixtures were dried at 120 °C and a portion of each mixture was sampled for thermogravimetric analysis (TGA). Then, the dried mixtures were divided into two batches and calcined at 850 and 950 °C for 4 h. The calcined powders were subjected to the second ball milling with identical conditions to the first one. The milled powders were again dried and compacted into pellets with 10 mm in diameter and 1.5 mm in thickness under a uniaxial pressure of 150 MPa, followed by cold isostatic pressing under 200 MPa for 2 min. Finally, these green pellets were sintered in the air at 1100 °C for 4 h.

### Characterization of Synthesis Reaction and Microstructure

TGA was performed by using a synchronous thermal analyzer (NETZSCH STA449F3, NETZSCH GmbH, Germany) from 30 to 1200 °C, with a heating rate of 10 °C min^−1^. The crystallographic information of the calcined powders was examined by using an X‐ray diffractometer (Rigaku 2500, Rigaku, Japan) with Cu K*α* radiation. SEM (JSM‐6460LV, JEOL, Japan), EPMA (JXA8230, JEOL, Japan), and Cs‐corrected scanning transmission electron microscope (STEM) (HF5000, Hitachi, Japan) were utilized to characterize the chemical homogeneity and domain configuration in sintered ceramics at different scales. For SEM and EPMA measurement, the samples were mechanically polished but without being subjected to any chemical or thermal etching. For TEM measurement, the sample was mechanically grounded and subjected to ion‐beam milling. The domain configuration of sintered ceramics was also characterized by using an atomic force microscope (MFP‐3D, Asylum Research, USA) with the PFM module. The preparation of samples for PFM measurement was identical to that for SEM and EPMA measurement. Phase structure evolution was investigated by using temperature‐dependent Raman spectroscopy (HR800, Horiba, Japan). A 632 nm laser excitation was employed in the back‐scattered measurement with a spatial resolution ≈3 µm. The spot size should be able to include the core‐shell heterostructure. The phase structure evolution was also investigated by using temperature‐dependent XRD (D8 Advance, Bruker, Japan) with Cu K*α* radiation, which was equipped with a temperature controller (2404, Eurotherm, UK). The diffraction pattern was collected for every 20 °C increasing from room temperature to 400 °C. The diffraction patterns were analyzed by using Line‐Profile Analysis Software (LIPRAS).^[^ ^33^ ^]^

### Characterization of Electrical Properties

The as‐sintered pellets were ground to 1 mm in thickness, coated with silver paste on both surfaces, and then fired at 600 °C for 30 min to form electrodes. Ceramic samples were poled at 120 °C in a silicone oil bath with an applied electric field of 4 kV mm^−1^ for 30 min, and cooled down with field‐on until room temperature. Temperature‐dependent dielectric properties were measured by using an impedance analyzer (TH2827, Changzhou Tonghui Electronic Co, China), which was coupled with a temperature‐regulated furnace. The quasi‐static piezoelectric coefficient *d*_33_ was measured by using a Berlincourt piezo‐meter (ZJ‐3A, Institute of Acoustics, Chinese Academy of Science, China). For *d*_33_ measurement, three pellets of the same batch were measured for each system; the samples are aged for at least 24 h before measurement.

## Conflict of Interest

The authors declare no conflict of interest.

## Supporting information

Supporting information

## Data Availability

The data that support the findings of this study are available from the corresponding author upon reasonable request.
