# Supplementary material for: Domain Engineering in Bulk Ferroelectric Ceramics via Mesoscopic Chemical Inhomogeneity
Source: Adv Sci (Weinh). 2022 Apr 17;9(17):2200998. doi: 10.1002/advs.202200998 (PMC9189658; doi:10.1002/advs.202200998)
Supplement: Supplementary file 1 — Supporting information [file ADVS-9-2200998-s001.pdf]

ADVANCED SCIENCE
Open Access

Supporting Information

for *Adv. Sci.*, DOI 10.1002/advs.202200998

Domain Engineering in Bulk Ferroelectric Ceramics via Mesoscopic Chemical Inhomogeneity

Hao-Cheng Thong, Zhao Li, Jing-Tong Lu, Chen-Bo-Wen Li, Yi-Xuan Liu, Qiannan Sun, Zhengqian Fu, Yan Wei* and Ke Wang**

Supporting Information

Domain Engineering in Bulk Ferroelectric Ceramics via Mesoscopic Chemical Inhomogeneity

Hao-Cheng Thong[#]*, Zhao Li*[#]*, Jing-Tong Lu, Chen-Bo-Wen Li, Yi-Xuan Liu, Qiannan Sun, Zhengqian Fu*, Yan Wei*, and Ke Wang**

H.-C. Thong, Z. Li, J.-T. Lu, C.-B.-W. Li, Y.-X. Liu, and Prof. K. Wang
State Key Laboratory of New Ceramics and Fine Processing, School of Materials Science and Engineering, Tsinghua University, Beijing 100084, PR China.

Q. Sun, Prof. Y. Wei
Beijing Laboratory of Biomedical Materials, Department of Geriatric Dentistry, Peking University School and Hospital of Stomatology, Beijing 100081, P. R. China

Prof. Z. Fu
State Key Laboratory of High Performance Ceramics and Superfine Microstructures, Shanghai Institute of Ceramics, Chinese Academy of Sciences, Shanghai 200050, PR China.

E-mail: fmail600@mail.sic.ac.cn (Prof. Z. Fu), kqweiyan@bjmu.edu.cn (Prof. Y. Wei), wang-ke@tsinghua.edu.cn (Prof. K. Wang)

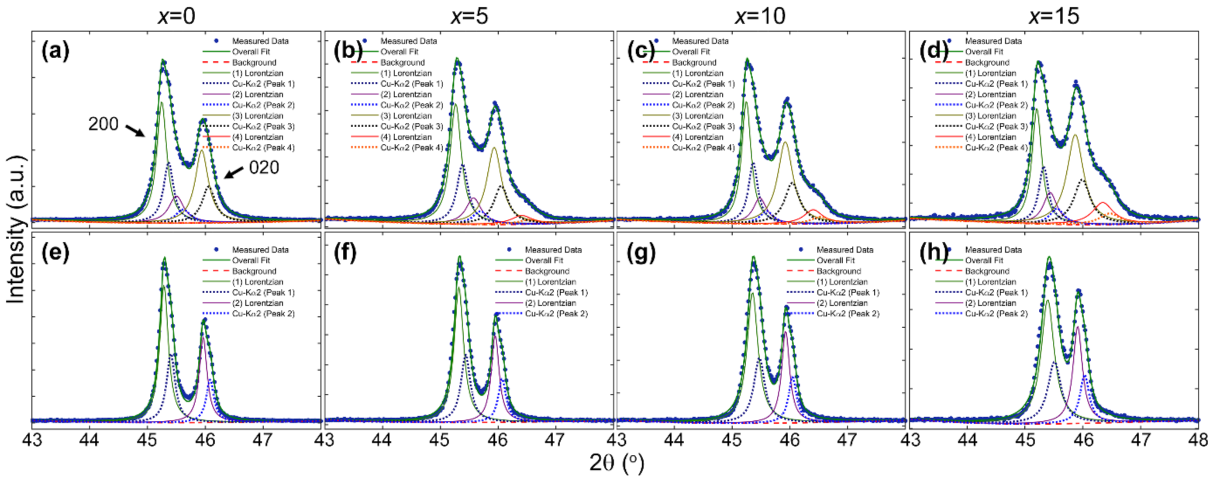

Figure S1. XRD analysis of the KNN-*x*Ta powders. (a) *x* = 0%, (b) *x* = 5%, (c) *x* = 10%, and (d) *x* = 15% powders calcined at 850 °C. (e) *x* = 0%, (f) *x* = 5%, (g) *x* = 10%, and (h) *x* = 15% powders calcined at 950 °C.

Please note that the assignment of primary phase and secondary phase is interchangeable, but for the sake of simplicity, the phase which possesses reflections of higher intensity is labeled as primary phase. Peaks (1) and (3) correspond to the (200)/(002) and (020) reflections of primary phase. Peaks (2) and (4) correspond to the (200)/(002) and (020) reflections of secondary phase. For powders calcined at 850 °C, the amount of secondary phase increases significantly with Ta addition. However, for powders calcined at 950 °C, no significant secondary phase is observed. Instead, the peaks of primary phase seem to shift towards each other, indicating the interdiffusion of primary phase and secondary phase.

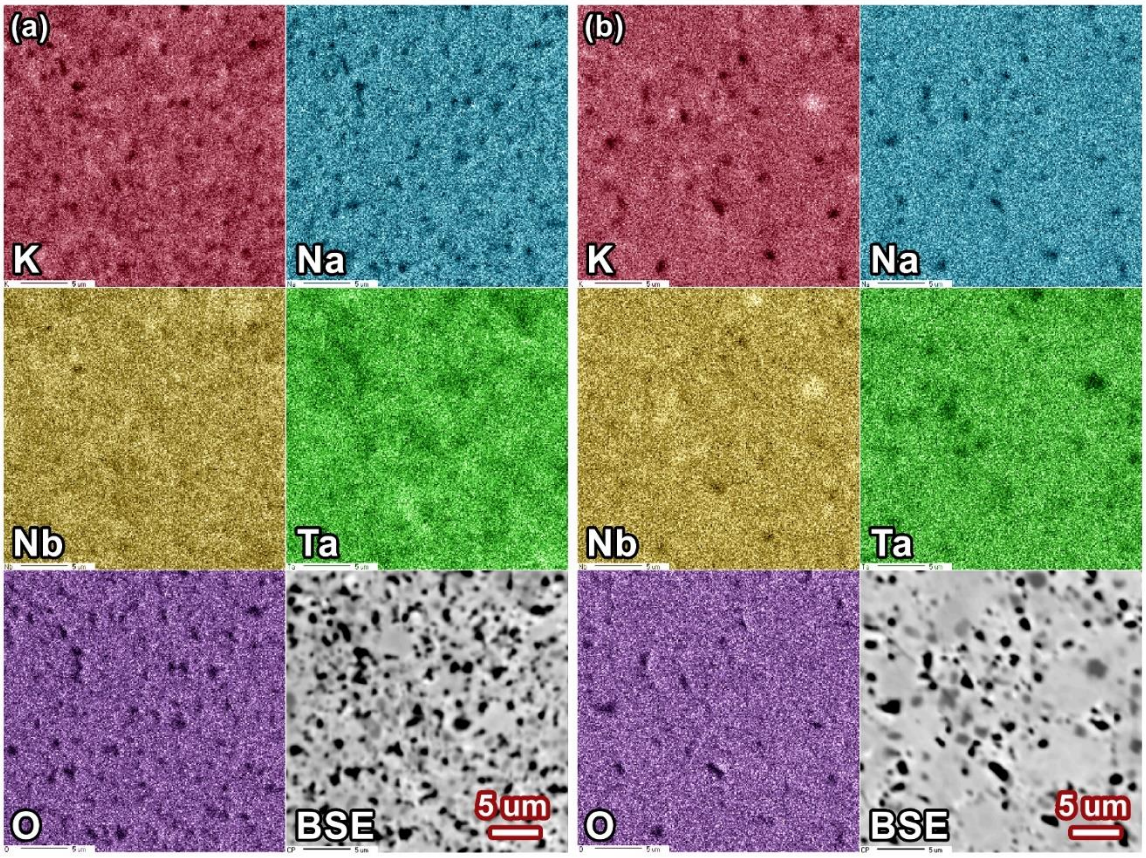

Figure S2. Electron probe microanalysis images of (a) KNN-15Ta@850 and (b) KNN-15Ta@950.

From Figure S2, we find that the chemical inhomogeneity in KNN-15Ta@850 is almost everywhere, compared to little but concentrated chemical inhomogeneity in KNN-15Ta@950. From Figure S2b, the distribution of Nb seems to compensate with that of Ta.

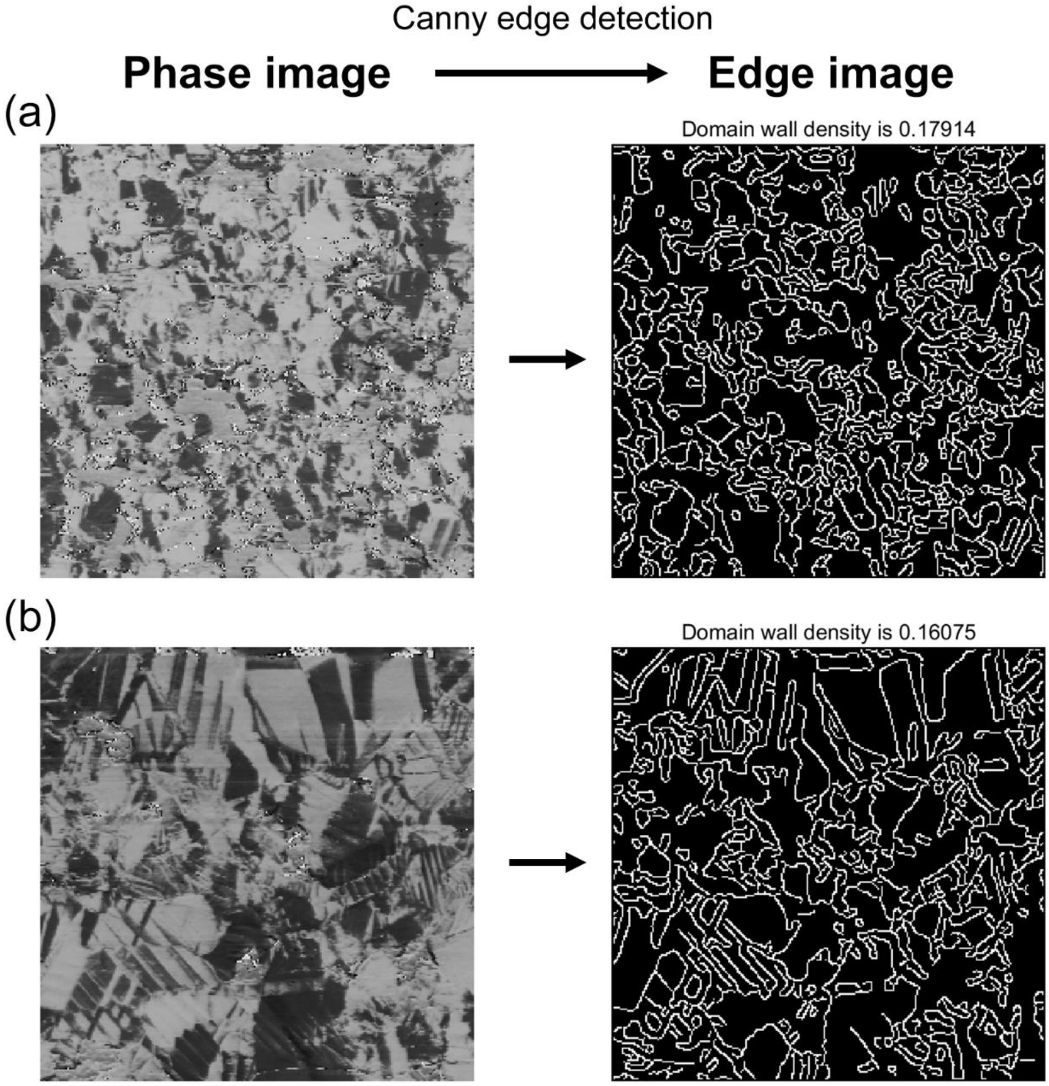

Figure S3. Detection of domain wall in KNN-15Ta@850 and (b) KNN-15Ta@950. The area of images is 10 μm x 10 μm.

The domain wall is semi-quantitatively evaluated from the edge detection of phase images. The edge detection was done by using Canny algorithm via programming software MATLAB. Even though the edge detection is visually satisfactory, the signal of grain boundaries can also be included along with the domain wall as the grain boundaries cannot be extracted accurately from the phase image. The domain wall density is estimated from the ratio of edge area (white pixels) over the total area (black and white pixels). Domain-wall densities of KNN-15Ta@850 (ratio=0.179) is roughly 10% higher than KNN-15Ta@950 (ratio=0.161).

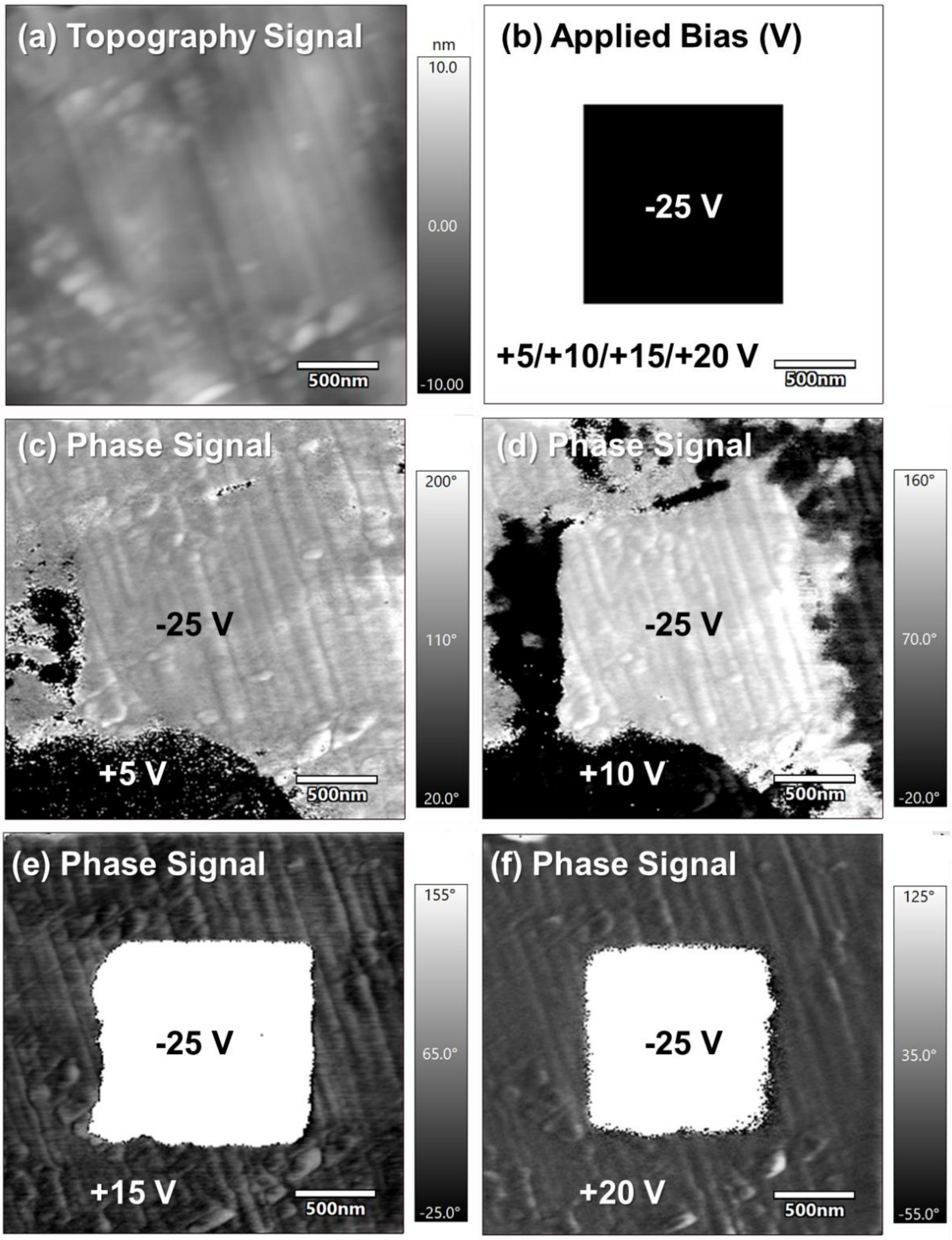

Figure S4. Direct writing of ferroelectric domain on KNN-15Ta@850. (a) Topography signal and (b) poling voltage applied on the selected 3 μm x 3 μm region. A negative voltage of 25 V was first applied on the whole region (black and white regions) before positive voltages were applied at the boundary (white region). Phase signals of the selected region when different positive voltages are applied, including (c) 5 V, (d) 10 V, (e) 15 V, (f) 20 V.

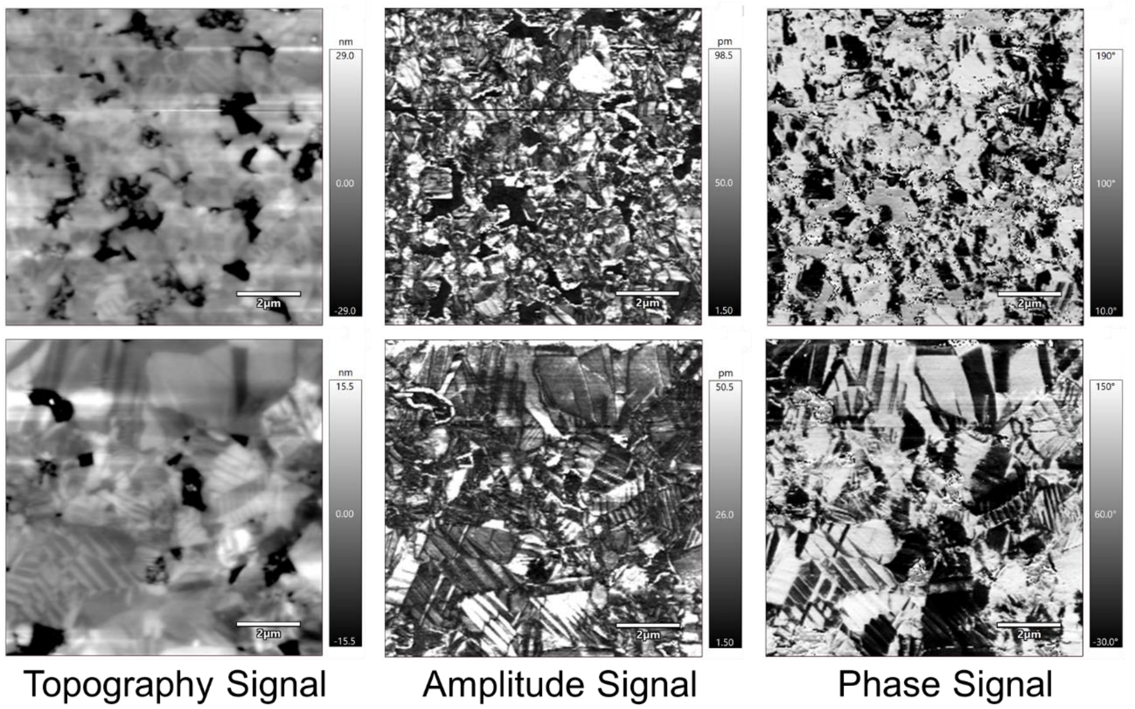

Figure S5. Original PFM data corresponds to PFM data presented in Figure 3.

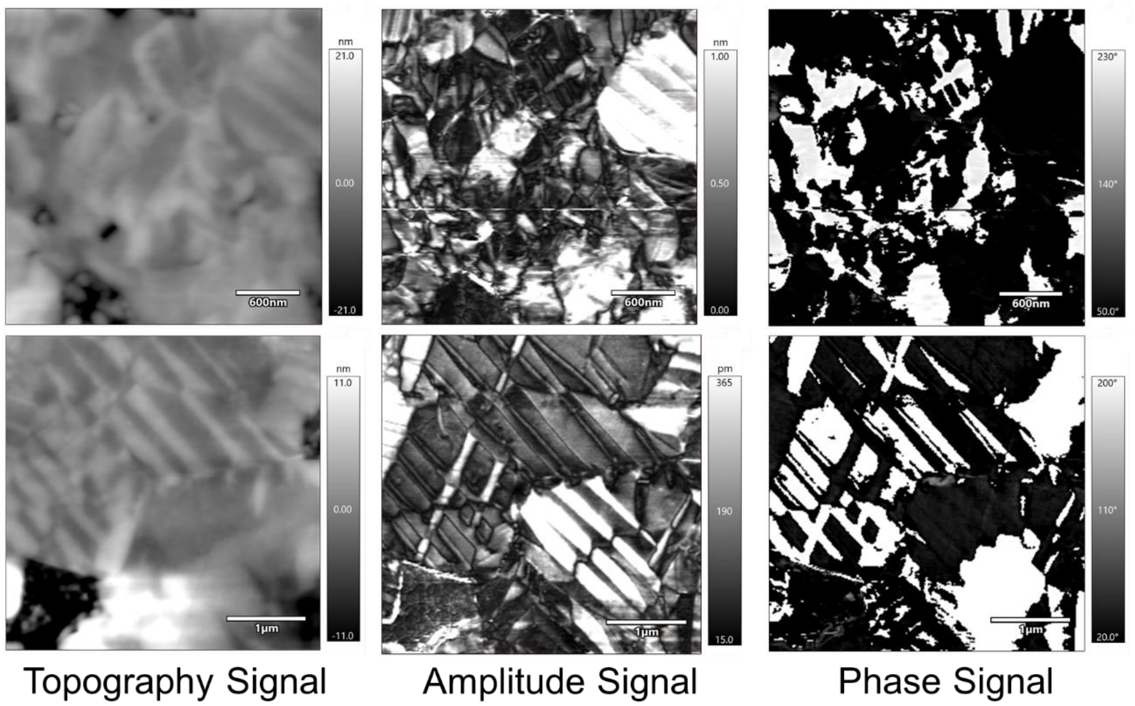

Figure S6. Original PFM data corresponds to PFM data presented in Figure 4.

Table S1. The skeletal density of sintered ceramic samples.

Density [g/cm^3]	x=0	x=5	x=10	x=15
850 ℃	4.15(2)	4.44(1)	4.45(2)	4.48(17)
950 ℃	4.34(2)	4.48(5)	4.43(7)	4.60(3)

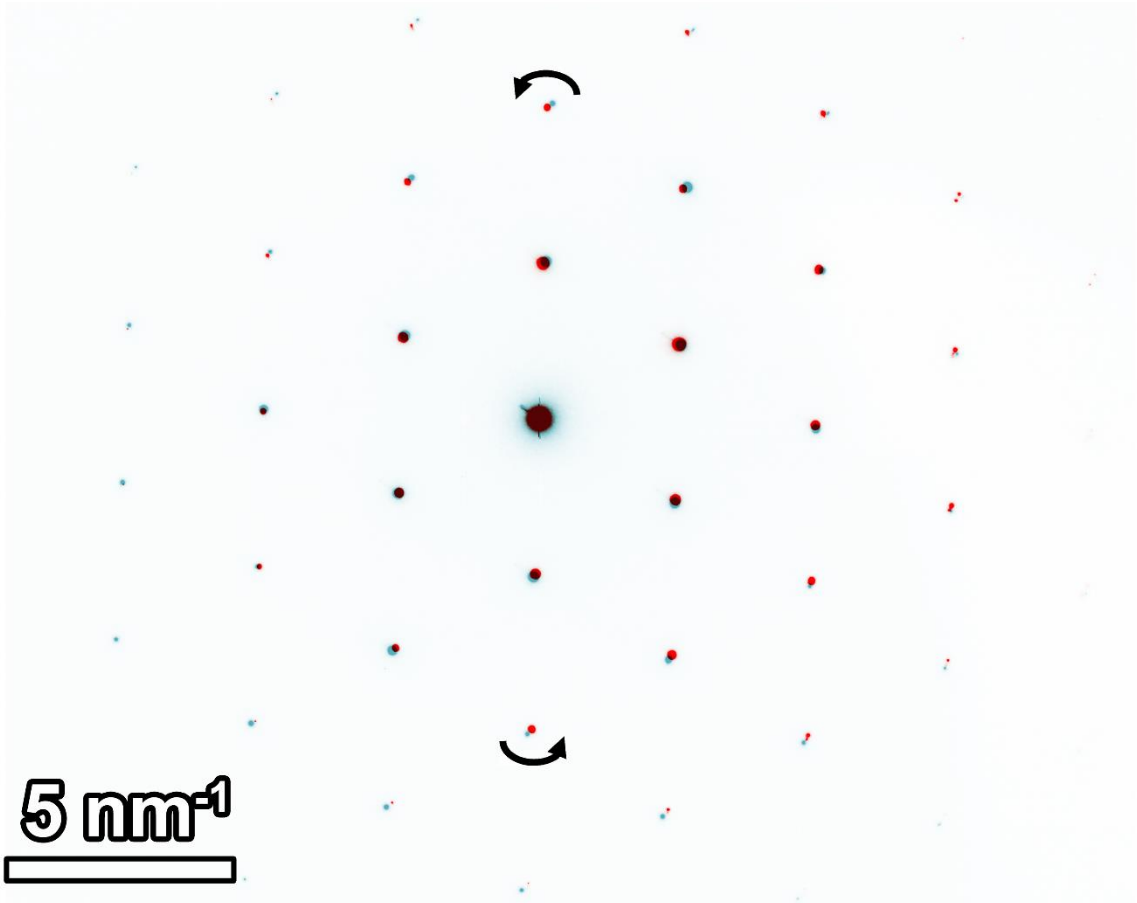

Figure S7. Overlapping SAED patterns of “core” (red spots) and “shell” (blue spots) region mentioned in Figure 5. The tilting direction of the “core” SAED pattern with respect to the “shell” SAED pattern was marked with black curved arrows.

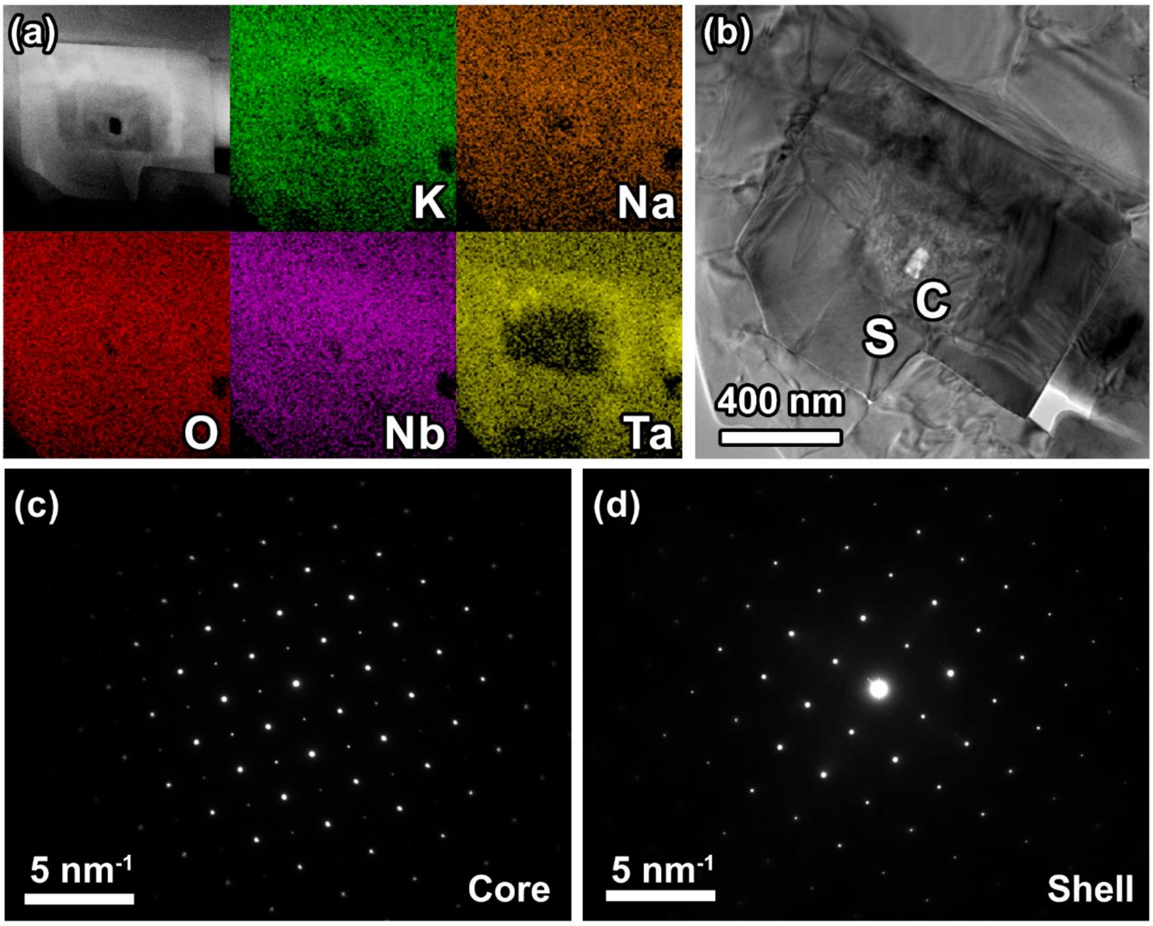

Figure S8. TEM analysis of the MCI regions in KNN-15Ta@850 polycrystalline ferroelectric. (a) EDS analysis and (b) bright-field TEM image of MCI region. SAED patterns of (c) "core" and (d) "shell" regions. The letters "C" and "S" represent "core" and "shell" respectively.

Reflections of superlattice can be found in the SAED pattern of "core", as shown in Figure S8c. Superlattice-reflection-filtered dark-field TEM is presented in Figure S9.

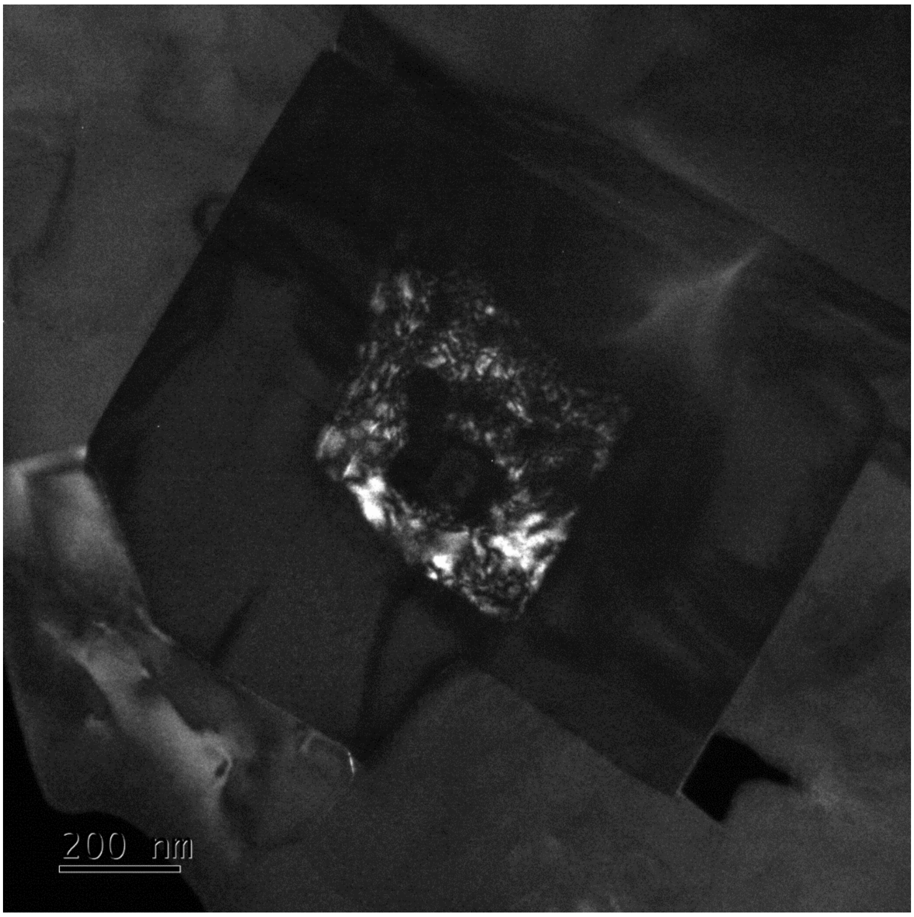

Figure S9. Superlattice-reflection-filtered dark-field TEM at MCI region. The bright area corresponds to the region consist of superlattices.

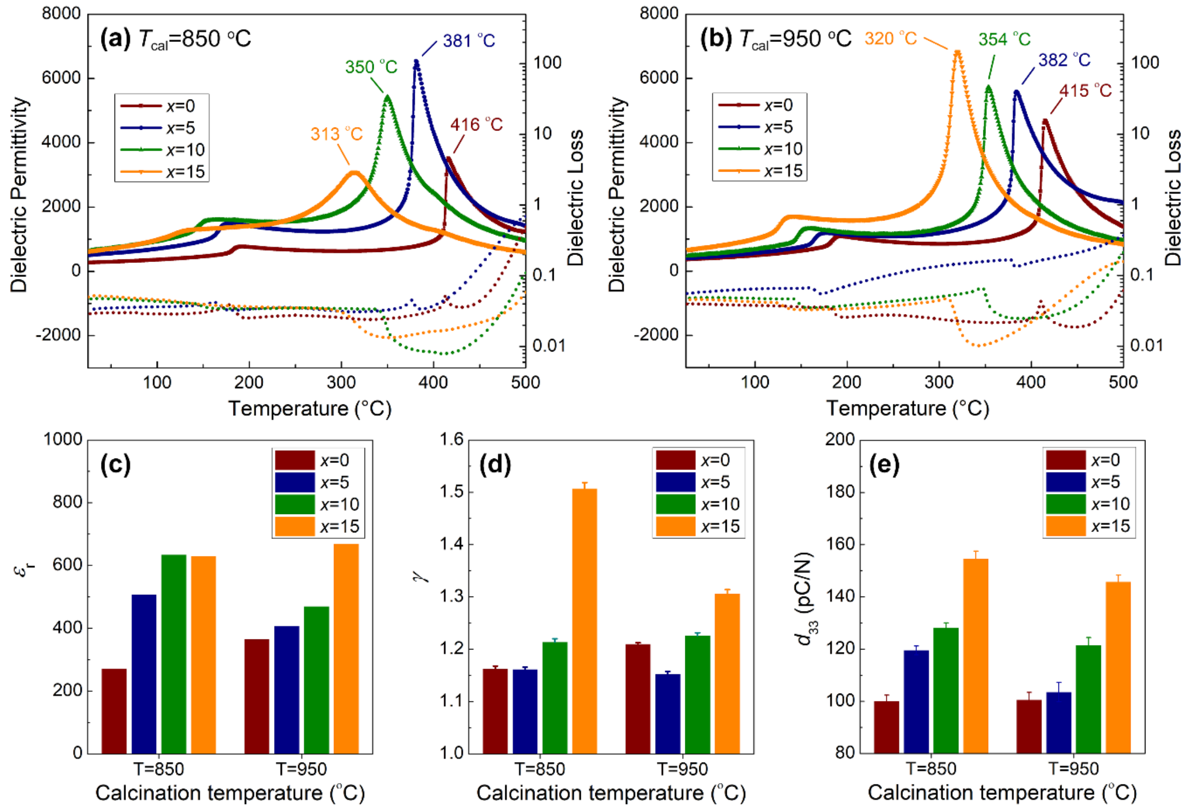

Figure S10. Comprehensive characterization of electrical properties. Temperature-dependent dielectric properties (measured at 100 kHz upon cooling) of KNN-*x*Ta ceramics sintered from powders calcined at (a) 850 ℃ and (b) 950 ℃. (c) dielectric permittivity at room temperature. (d) fitted γ of paraelectric-ferroelectric phase transition from the modified Curie-Weiss law. (e) Quasi-static d_{33} measured at room temperature.

The quasi-static d_{33} measured at room temperature is found to increase as a function of Ta addition, as shown in Figure S10e. It is worth noting that most d_{33} properties of samples sintered from the powder calcined at 850 ℃ are higher than those calcined at 950 ℃. The enhancement might originate from the facilitated extrinsic contribution due to the modified domain configuration (*i.e.*, increased domain-wall density). However, since d_{33} can be affected by many factors, including porosity, defects, *etc*., conservatively, we should not link the promoted piezoelectricity with the engineered domain configuration herein, but recommend further investigation.

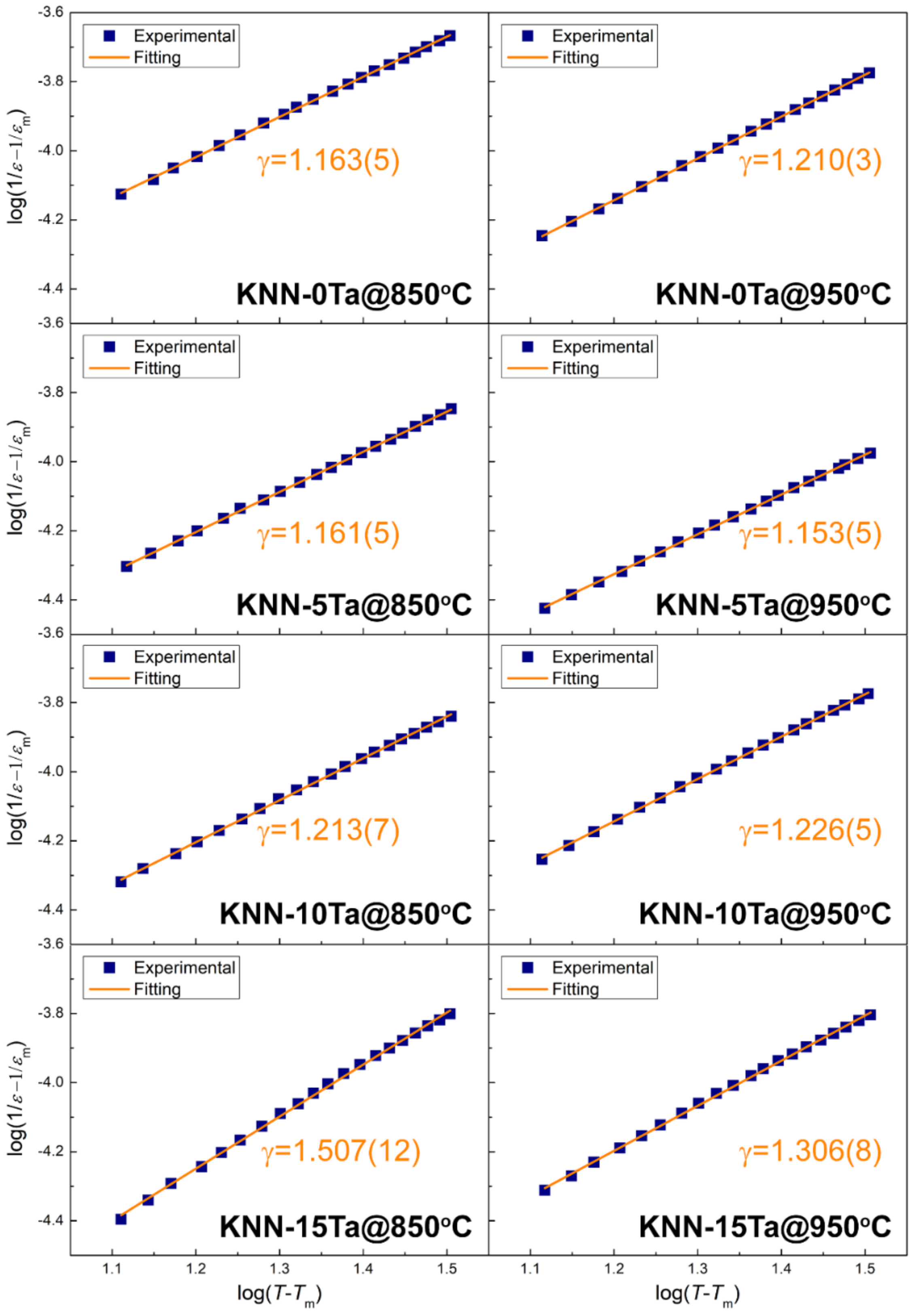

Figure S11. Fitting of diffused phase transition in KNN-*x*Ta sintered ceramics by using modified Curie-Weiss law.

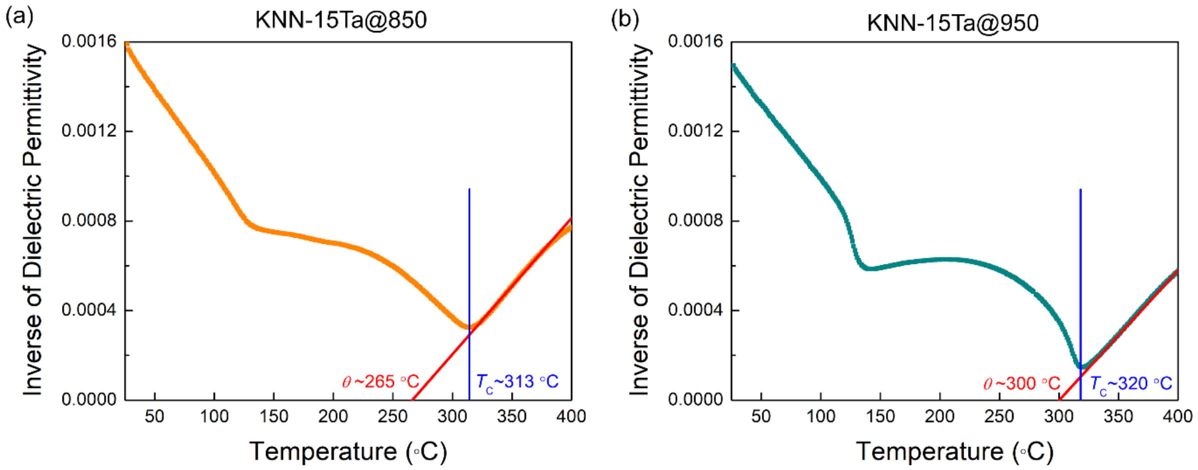

Figure S12. Analysis of the second-order nature of the ferroelectric-paraelectric phase transition in (a) KNN-15Ta@850 and (b) KNN-15Ta@950.

The first-order nature of phase transition can be inferred from the difference between Curie-Weiss temperature (θ) and Curie Temperature (T_C), *i.e.*, the greater the difference, the stronger the first-order nature. From Figure S12, it is observed that KNN-15Ta@850 shows a stronger first-order nature than KNN-15Ta@950.

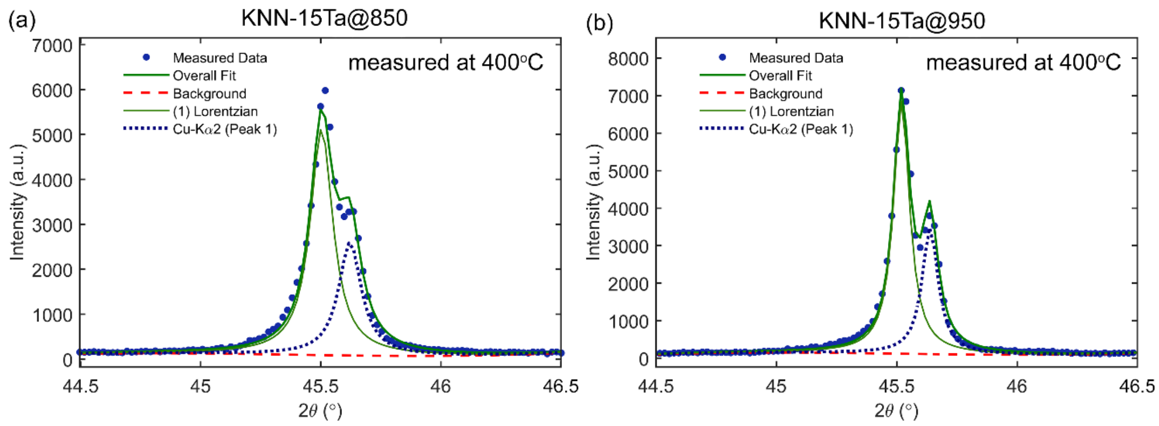

Figure S13. Fitting of 200_C reflections of (a) KNN-15Ta@850 and (b) KNN-15Ta@950.

Table S2. Summary of fitting results displayed in Figure S13.

Sample	Peak center	Peak center error	FWHM	FWHM Error	%Rwp
KNN-15Ta@850	45.503	0.002	0.115	0.006	9.823
KNN-15Ta@950	45.520	0.002	0.081	0.004	10.089

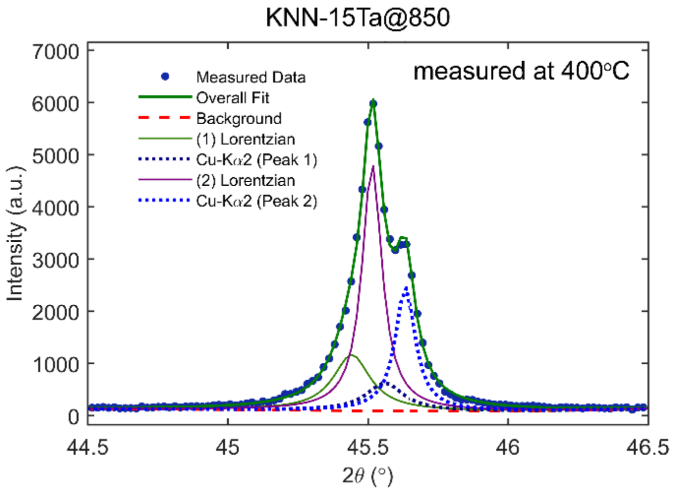

Figure S14. Fitting of 200_C reflections of KNN-15Ta@850 using 2 peak functions.

From Figure S13, it is observed that the diffraction peak of KNN-15Ta@850 is broader than that of the KNN-15Ta@950, as supported by the analysis of FWHM in Table S2. From Figure S14, we find that fitting using 2 peak functions shows better agreement with the experimental diffraction pattern.

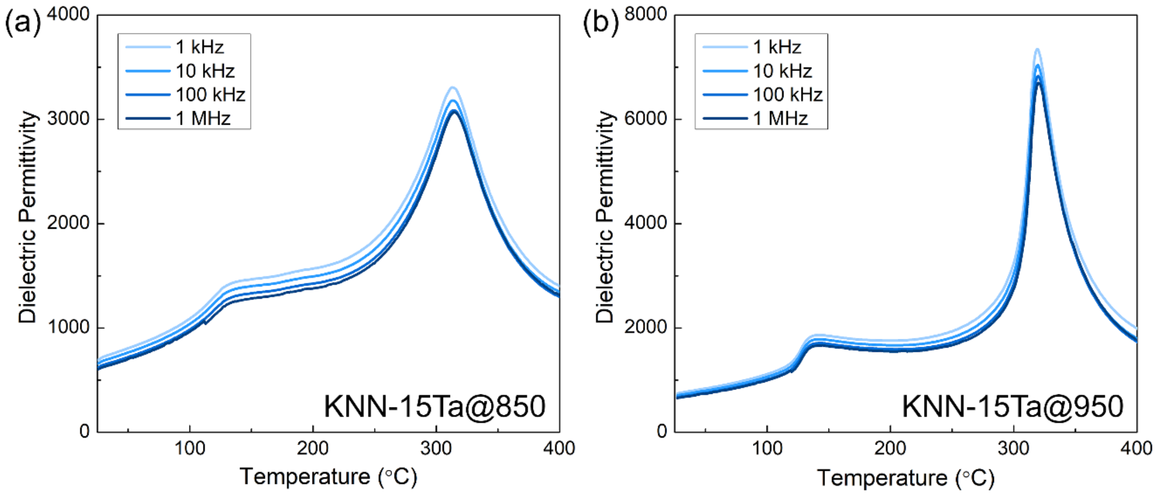

Figure S15. Temperature-dependent dielectric spectra of (a) KNN-15Ta@850 and (b) KNN-15Ta@950 samples measured as a function of frequency.

It is observed that the dielectric permittivity decreases with increasing frequency, which is likely due to the diminishing space-charge polarization mechanism at high frequency. No frequency dispersion in a typical relaxor, *i.e.*, the shift of T_C as a function of increasing frequency, is observed for both samples.
